# Colchicine Alleviates Cholesterol Crystal-Induced Endothelial Cell Pyroptosis through Activating AMPK/SIRT1 Pathway

**DOI:** 10.1155/2020/9173530

**Published:** 2020-07-15

**Authors:** Mengyue Yang, Hang Lv, Qi Liu, Lu Zhang, Ruoxi Zhang, Xingtao Huang, Xuedong Wang, Baihe Han, Shenglong Hou, Dandan Liu, Gang Wang, Jingbo Hou, Bo Yu

**Affiliations:** ^1^The Key Laboratory of Myocardial Ischemia Organization, Chinese Ministry of Education, Harbin, Heilongjiang 150086, China; ^2^Department of Cardiology Organization, The Second Affiliated Hospital of Harbin Medical University, Harbin, Heilongjiang 150086, China; ^3^Department of Cardiology, The Affiliated Cardiovascular Hospital of Xiamen University, Xiamen, Fujian 316006, China

## Abstract

Cholesterol crystal- (CC-) induced endothelial cell inflammation and pyroptosis play an important role in the development of cardiovascular diseases, especially in atherosclerosis (AS). Increasing evidence suggests that cholesterol crystals are known to be a pivotal pathological marker of atherosclerotic plaque vulnerability. As a classical nonspecific anti-inflammatory drug, colchicine has been widely used in the treatment of acute gout. However, whether colchicine could alleviate CC-induced endothelial cell injury and the related mechanisms remains to be addressed. In this study, the protective effect of colchicine on human umbilical vein endothelial cells (HUVECs) was confirmed. Our results revealed that after cotreatment with colchicine and cholesterol crystals in endothelial cells, the uptake of cholesterol crystals was significantly decreased, the cell viability was obviously increased, and the release of lactate dehydrogenase (LDH) and the number of pyroptotic cells decreased significantly; then, the expression of NLRP3 inflammasome-related proteins and various inflammatory factors was also visibly suppressed; moreover, as a potent activator of NLRP3 inflammasome, the intracellular ROS level was clearly reduced, while mitochondrial membrane potential improved significantly. In addition, the expression levels of AMP-dependent kinase (AMPK) pathway-related proteins as well as various antioxidant enzymes were elevated notably in varying degrees. However, the above effects of colchicine were completely offset by the treatment of siRNA targeting AMPK*α* and Sirtuin1 (SIRT1). Therefore, we conclude that colchicine plays a crucial role in alleviating the intracellular inflammatory response and NLRP3 inflammation activation, attenuating the levels of cellular oxidative stress and pyroptosis in endothelial cells via regulating AMPK/SIRT1 signaling, which may be a concrete mechanism for the secondary prevention of cardiovascular diseases.

## 1. Introduction

Coronary heart disease (CHD) is the most fatal disease in the world, and acute coronary syndrome (ACS) remains a leading cause of morbidity and mortality. Accordingly, vulnerable plaque is the potential culprit of ACS [1]. Pathological studies have shown that the more CC content in atherosclerotic plaque, the faster the plaque progresses and the more prone to rupture or erosion leading to unstable cardiovascular events [2, 3]. Therefore, CC is a pivotal pathological marker of plaque vulnerability.

Extensive studies have found that CC appeared in the initiation of atherosclerotic plaque and was associated with early inflammatory response [2]. CC could activate NLRP3 inflammasome, induce local inflammation, and promote the formation of large necrotic cores and vulnerable plaque [1]. NLRP3 inflammasome is a macromolecule-polyprotein complex that regulates the production of the IL-1 family and plays an important role in the pathogenesis of AS. It can be activated by a variety of damaging molecules such as ATP, uric acid crystal, cholesterol crystal, and asbestos, triggering an intensively aseptic inflammatory response via upregulating the expression of multiple proinflammatory cytokines [4]; beyond that, pyroptosis implementing protein GSDMD is cleaved by activated caspase-1, inducing caspase-1-dependent pyroptotic cell death [5]. Moreover, studies have demonstrated that reactive oxygen species (ROS) plays a crucial role in the activation of inflammasome, and pretreatment with various ROS scavengers represses NLRP3 inflammasome activation in response to a series of agonists [6, 7].

Pyroptosis is a newly discovered type of programmed cell death accompanied by inflammatory response. Recent studies have reported that inflammation and pyroptosis play an important role in the progression of cardiovascular diseases, such as atherosclerosis, myocardial infarction and ischemia-reperfusion injury, diabetic cardiomyopathy, and heart failure [8–11]. Different from apoptosis or necroptosis, pyroptotic cells are manifested as the formation of a large number of protein holes on the cell membrane, resulting in the rapid loss of cell membrane integrity and the significant weakening of the ability to regulate the flow of substances, thus leading to the release of proinflammatory substances and the enlarged secondary inflammation [12]. Therefore, cell pyroptosis may play a prominent role in AS-interrelated inflammation, and targeted regulation of pyroptotic cells in atherosclerotic lesions may be a new direction for the treatment of AS.

Colchicine is a classic treatment for acute gout attacks. Recent studies have shown that low-dose colchicine can also be used for the secondary prevention of cardiovascular diseases due to its powerful anti-inflammatory effect [13]. The latest clinical study published in the *New England Journal of Medicine* also showed that in the assessment of 4745 patients with recent myocardial infarction, taking low-dose colchicine daily (0.5 mg) significantly reduced the risk of ischemic cardiovascular events compared with the placebo group [14]. Related research has indicated that colchicine could inhibit macrophage phenotypic switch induced by monosodium urate crystals and alleviate macrophage inflammation via activating the LKB1-AMPK pathway [15]. Moreover, activated AMPK could enhance the expression level and deacetylation activity of SIRT1 by promoting the production of NAD+, the SIRT1 activator. Furthermore, the activation of SIRT1 could catalyze the deacetylation of peroxisome proliferator-activated receptor *γ* coactivator 1*α* (PGC-1*α*), then regulate the expression of various antioxidant enzymes, subsequently following the deceleration of the intracellular ROS level and oxidative stress [16]. However, it is unclear whether colchicine could suppress the activation of NLRP3 inflammasome and cell pyroptosis induced by CC in the progression of AS, and direct evidence for AMPK/SIRT1 activation in antagonizing inflammasome and pyroptosis is still lacking.

In this study, we discovered that colchicine could upregulate the expression and activity of various antioxidant enzymes, inhibit ROS production and NLRP3 inflammasome activation, and antagonize endothelial cell pyroptosis which was induced by CC via activating the AMPK-SIRT1 pathway.

## 2. Materials and Methods

### 2.1. Cell Culture and Reagent Treatments

Human umbilical vein endothelial cells (HUVECs) were obtained from Science Cell Research Laboratories (Carlsbad, CA, USA) and cultured in Endothelial Cell Medium (ECM, Science Cell Research Laboratories, Carlsbad, CA, USA) supplemented with 1% endothelial cell growth factors, 5% FBS, and 1% penicillin/streptomycin at 37°C in a 5% CO_2_ humidified atmosphere. Cholesterol (C3045) and total ROS scavenger N-acetyl-L-cysteine (NAC, A7250) were purchased from Sigma-Aldrich; colchicine (HY-16569) was obtained from MCE. For experiments involving pharmacological reagents, endothelial cells were pretreated with colchicine (0-100 nM) or NAC (5 mM) for 2 h and subsequently followed by cholesterol crystals (0.5 mg/ml) for 10-24 h in the presence of these reagents.

### 2.2. Preparation of Cholesterol Crystals

100 mg ultrapure cholesterol powder (Sigma, Saint Louis, MO) dissolved in 8 ml 95% ethanol was heated to 60°C, filtered through Whatman filter paper while still warm, and left at room temperature to allow crystallization to proceed. After drying, relatively large cholesterol crystals were developed and ground with a grinding bowl to make the crystal size within 1-10 *μ*m. The collected crystals were sterilized by autoclaving and ultraviolet light, then stored at −20°C for reserve. Cholesterol crystals were used at a final concentration of 0.5 mg/ml if not otherwise specified.

### 2.3. Cell Viability Assay

A CCK8 assay was performed to evaluate cell viability of HUVECs following the manufacturer's instruction (CK04, Dojindo, Kumamoto, Japan). 2 × 10^3^ endothelial cells were seeded in 96-well plates in complete medium and treated with different concentrations of colchicine and/or cholesterol crystals for 24 h. Afterwards, a 10 *μ*l CCK8 reagent was added to each well, and the cells were incubated for 3 h at 37°C. Absorbance measurements were taken at 450 nm using a Tecan Infinite M200 microplate reader (LabX, Austria).

### 2.4. Hoechst 33342/PI Fluorescent Staining

To assess pyroptosis, cells were double-stained with Hoechst 33342 and propidium iodide (PI). HUVECs (10^5^ cells/well) were cultured in a 12-well plate and were pretreated with test drugs or siRNA for the duration as to be specified in the appropriate section. After treatments, the cells in each group were washed with PBS for three times and stained with 2.5 *μ*l Hoechst 33342 and 2.5 *μ*l PI (C1056, Beyotime Institute of Biotechnology, Shanghai, China) for 30 min at 4°C in the dark. The stained cells were examined under a fluorescence microscope at 200x magnification (DMI4000B; Leica, Wetzlar, Germany).

### 2.5. Transmission Electron Microscopy (TEM)

The samples were fixed with 2.5% glutaraldehyde overnight at 4°C and 1% osmium acid for 1.5 h, then rinsed with PBS three times for 15 min. Endothelial cells were dehydrated with ethanol at concentration gradients (30%, 50%, 70%, 80%, 90%, 95%, and 100%) for 15 minutes at a time, then embedded with a mixture of the embedding agent and acetone overnight at 70°C. 70-90 nm sections were obtained with Reichert-Jung Ultracut E Ultramicrotome and stained with lead citrate and uranyl acetate. Finally, the Hitachi H-7650 transmission electron microscope was used for observation (Hitachi 7650, Japan).

### 2.6. Immunofluorescent Assay

To detect the activation of NLRP3, endothelial cells were fixed and permeated at room temperature, then incubated with primary antibody against NLRP3 (diluted in goat serum, 1 : 100) overnight at 4°C. Afterwards, the cells were flushed and incubated with fluorescent secondary antibody diluted with goat serum (1 : 500) at room temperature for 60 min in the dark. Finally, DAPI was used to stain the cell nucleus for 10 min. The cells were viewed and photographed on a confocal laser microscope at 400x magnification (LSM 800, ZEISS, Germany).

### 2.7. Measurement of Reactive Oxygen Species (ROS)

The accumulation of cellular ROS was detected by the fluorescence probe 2′,7′-dichlorofluorescein diacetate (DCFH-DA, D6883, Sigma-Aldrich, MO, USA). HUVECs cultured in 24-well plates were loaded with DCFH-DA (10 *μ*M) in the dark for 20 min at 37°C, followed by washing with serum-free medium three times. Fluorescence was observed with the fluorescent microscope.

### 2.8. Detection of Mitochondrial Membrane Potential

The mitochondrial membrane potential was measured by the fluorescence probe JC-1 using the mitochondrial membrane potential assay kit (C2006, Beyotime Institute of Biotechnology). HUVECs (2 × 10^4^ cells/well) were seeded in a 24-well plate, while the cells were treated according to the corresponding conditions; the culture medium was absorbed, and the cells were washed with PBS once. Then, 500 *μ*l culture medium was added to the plate, which was followed by 500 *μ*l JC-1 dyeing solution. The endothelial cells were incubated for 20 minutes at 37°C in the dark, then washed with JC-1 staining buffer (1x) twice. Fluorescence was observed under a fluorescence microscope.

### 2.9. Cytotoxicity Assay

Relevant endothelial cells were treated as indicated. Cytotoxicity was determined by measuring the lactate dehydrogenase (LDH) released from cells, using the LDH assay kit (A020, Nanjing Jiancheng Biology Engineering Institute, Nanjing, Jiangsu, China) according to the manufacturer's instruction. The absorbance was determined at a wavelength of 450 nm on a spectrophotometric microplate reader.

### 2.10. Lipid Peroxidation MDA Assay

Lipid peroxidation occurs when cells undergo oxidative stress. Therefore, the lipid peroxidation MDA assay kit (S0131, Beyotime Institute of Biotechnology) was used to detect the content of MDA in HUVECs following the manufacturer's instruction. Absorbance measurements were taken at 532 nm.

### 2.11. GSSG Content Detection

Oxidized glutathione disulfide (GSSG) increased significantly in oxidative stress. To test the endothelial cell GSSG content, the GSH and GSSG assay kit was taken (S0053, Beyotime Institute of Biotechnology) according to the manufacturer's instruction.

### 2.12. siRNA Transfection

Small interfering RNAs (siRNAs) of AMPK and SIRT1 and the negative control were synthesized by RiboBio (Guangzhou, China). HUVECs were seeded on 12-well plates (10^5^ cells/well); upon reaching 40% confluency, the cells were transfected with 50 *μ*M siRNA using the riboFECT™ CP Reagent (RiboBio, Guangzhou, China) according to the manufacturer's instructions. After 48 h of incubation with the siRNAs, endothelial cells were exposed to colchicine (10 nM) and cholesterol crystals (0.5 mg/ml) for 24 h and then collected for quantitative real-time PCR and Western blotting.

### 2.13. RNA Isolation and RT-PCR

The TRIZOL reagent (15596018, Invitrogen, CA, USA) was applied to extract total RNA from HUVECs. cDNA was synthesized from 1 *μ*g of total RNA using the iScript gDNA Clear cDNA Synthesis Kit (170-8890, Bio-Rad Laboratories, Redmond, USA). Quantitative RT-PCR was performed using a SsoFast EvaGreen Supermix (172-5260, Bio-Rad Laboratories) on a CFX96 Real-Time PCR Detection System (Bio-Rad Laboratories) following the manufacturer's protocol. The following optimized conditions were used: 95°C for 30 s, 95°C for 5 s, and 40 cycles at 60°C for 5 s. The levels of mRNA were normalized in relevance to endogenous GAPDH, and the expression of target genes was analyzed by the method of 2^-*ΔΔ*ct^. The sequence of related genes is shown as follows: AMPK*α*: forward: 5′-ACCAAGGGCACGCCATAC-3′, reverse: 5′-TCTTCCTTCGTACACGCAAA-3′; SIRT1: forward: 5′-TAGCCTTGTCAGATAAGGAAGGA-3′, reverse: 5′-ACAGCTTCACAGTCAACTTTGT-3′; GSDMD: forward: 5′-GTGTGTCAACCTGTCTATCAAGG-3′, reverse: 5′-CATGGCATCGTAGAAGTGGAAG-3′; IL-18: forward: 5′-TCTTCATTGACCAAGGAAATCGG-3′, reverse: 5′-TCCGGGGTGCATTATCTCTAC-3′; IL-1*β*: forward: 5′-ATGATGGCTTATTACAGTGGCAA-3′, reverse: 5′-GTCGGAGATTCGTAGCTGGA-3′; IL-6: forward: 5′-ACTCACCTCTTCAGAACGAATTG-3′, reverse: 5′-CCATCTTTGGAAGGTTCAGGTTG-3′; IL-8: forward: 5′-ACTGAGAGTGATTGAGAGTGGAC-3′, reverse: 5′-AACCCTCTGCACCCAGTTTTC-3′; MCP-1: forward: 5′-CAGCCAGATGCAATCAATGCC-3′, reverse: 5′-TGGAATCCTGAACCCACTTCT-3′; and GAPDH: forward: 5′-CCACTCCTCCACCTTTGAC-3′, reverse: 5′-ACCCTGTTGCTGTAGCCA-3′.

### 2.14. Western Blotting Analysis

Protein extracts from 2 × 10^6^ endothelial cells were separated by 10% sodium dodecyl sulfate-polyacrylamide gel electrophoresis (SDS-PAGE) and transferred to polyvinylidene fluoride (PVDF) membranes (ISEQ00010, Millipore, Billerica, MA, USA) using a semidry transblot apparatus (Bio-Rad Laboratories, Redmond, USA). Subsequently, membranes were blocked with 5% nonfat dried milk (R&D Systems, Minneapolis, MN, USA) in Tris-buffered saline-Tween 20 (TBST) for 1 h at room temperature and then probed with specific primary mouse or rabbit antibodies against NLRP3 (Cell Signaling Technology (CST), Danvers, MA, USA, Cat. No.: 15101), ASC (Abcam, Cambridge, MA, USA, Cat. No.: ab70627), procaspase-1 (CST, Cat. No.: 3866S), caspase-1 (CST, Cat. No.: 4199S), AMPK (CST, Cat. No.: 2532S), p-AMPK (CST, Cat. No.: 2535S), SIRT1 (CST, Cat. No.: 2310S), HO-1 (Abcam, Cat. No.: ab68477), SOD-1 (Abcam, Cat. No.: ab13498), SOD-2 (CST, Cat. No.: 13194s), IL-18 (CST, Cat. No.: 54943S), IL-1*β* (CST, Cat. No.: 83186S), eNOS (CST, Cat. No.: 9572S), p-eNOS (CST, Cat. No.: 9571S), and *β*-actin (TA-09, Zhongshanjinqiao, Inc., Beijing, China) overnight at 4°C. After washing with TBST for three times, the membranes were incubated with the peroxidase-conjugated second antibody (ZB-2301/ZB-2305, Zhongshanjinqiao) for 1 h at room temperature. The immunoreactive bands were detected by chemiluminescence methods and visualized using Luminescent Imaging Workstation (Tanon, Shanghai, China; 6600), and the relative intensity was measured and analyzed using ImageJ software.

### 2.15. Statistical Analysis

All statistical analyses were performed with GraphPad Prism 8.0 software (GraphPad Software, San Diego, CA, USA) and were presented as the mean ± SD. Differences among groups were determined using a one-way ANOVA test. Each experiment was repeated at least three times, and *P* values < 0.05 were considered statistically significant.

## 3. Results

### 3.1. Colchicine Suppresses Cholesterol Crystal-Induced Endothelial Cell Pyroptosis

**Figure 1:**
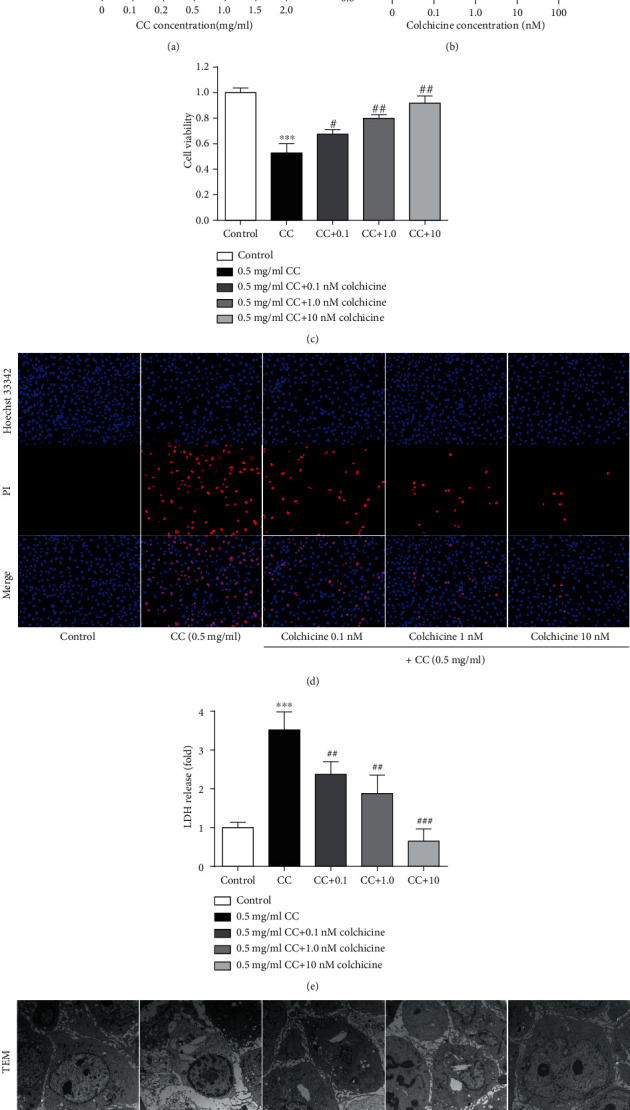
Colchicine suppresses cholesterol crystal-induced endothelial cell pyroptosis. (a–c) Impacts of increasing cholesterol crystals and colchicine concentrations on the viability of HUVECs. Endothelial cells treated with increasing doses of (a) cholesterol crystals (0-2.0 mg/ml) and (b) colchicine (0-100 nM) and (c) cholesterol crystal (0.5 mg/ml) and different concentrations of colchicine (0-10 nM) for 24 h. The cell viability was detected using a CCK8 assay. (d) Pyroptotic cell death was measured with Hoechst 33342 (blue)/PI (red) double-fluorescent staining (scale bars = 100 *μ*m). (e) The LDH release was evaluated with a cytotoxicity detection LDH kit. (f) Transmission electron microscopy (TEM) was used to observe the pyroptotic cell morphology (scale bar TEM = 2 *μ*m). Data was expressed as the mean ± SD of three separate experiments. ^∗∗^*P* < 0.01, ^∗∗∗^*P* < 0.001, and ^∗∗∗∗^*P* < 0.0001 vs. the control group. ^#^*P* < 0.05, ^##^*P* < 0.01, and ^###^*P* < 0.001 vs. the cholesterol crystal group.

A previous study has demonstrated that long-term use of low-dose colchicine could improve clinical outcomes in patients with advanced vascular disease by targeting an inflammatory pathway [13]. To examine the possible impact of colchicine on the inflammation of the vascular wall, we firstly explored whether pretreatment with colchicine could inhibit cholesterol crystal-induced endothelial cell pyroptosis. Consistent with a previous report [6], using the CCK8 assay, we found that the optimum dose of cholesterol crystals to induce pyroptosis was 0.5 mg/ml (Figure 1(a)). The cell viability was measured after incubation with various concentrations of colchicine (0-100 nM), and concentrations above 100 nM had an obvious toxic effect (Figure 1(b)). After being exposed to the cotreatment of cholesterol crystals and colchicine, pyroptotic cell death was evaluated with LDH released and Hoechst 33342/PI staining. In addition, cell survival was also detected. CC added to HUVECs significantly increased the release of LDH (Figure 1(e)), and the proportion of PI-positive staining cells was remarkably improved (Figure 1(d)). However, this phenomenon was counteracted by cotreatment with colchicine in a concentration-dependent manner (Figures 1(d)–1(e)). On the other hand, the cell viability decreased by CC was obviously elevated after colchicine intervention (Figure 1(c)). Furthermore, the results of transmission electron microscopy showed that with the increase of colchicine concentration, the cholesterol crystal uptake by endothelial cells decreased gradually, and the number of pyroptotic endothelial cells reduced observably, especially at the concentration of 10 nM (Figure 1(f)).

### 3.2. Colchicine Alleviates NLRP3 Inflammasome Activation Relevant in Pyroptosis

**Figure 2:**
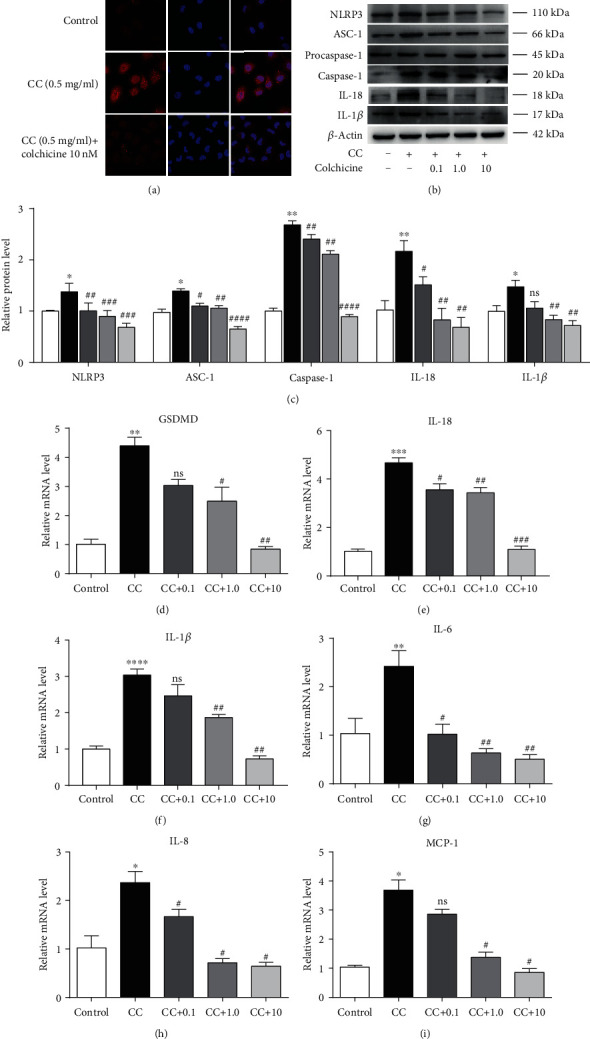
Colchicine alleviates NLRP3 inflammasome activation relevant in pyroptosis. HUVECs were treated for 24 h with different doses of colchicine (0–10 nM) in the presence of cholesterol crystal (0.5 mg/ml) or were left untreated (control). (a) Immunofluorescence was used for qualitative detection of NLRP3 (400x). (b) Western blotting was dedicated to examine the protein expression levels of NLRP3, ASC-1, procaspase-1, caspase-1, IL-18, and IL-1*β*. (c) Quantitative analysis of pyroptosis-associated protein expression. (d–i) Real-time PCR analysis of the mRNA levels of GSDMD and proinflammatory cytokines (IL-18, IL-1*β*, IL-6, IL-8, and MCP-1). Data was expressed as the mean ± SD of three separate experiments. ^∗^*P* < 0.05, ^∗∗^*P* < 0.01, ^∗∗∗^*P* < 0.001, and ^∗∗∗∗^*P* < 0.0001 vs. the control group. ^#^*P* < 0.05, ^##^*P* < 0.01, ^###^*P* < 0.001, and ^####^*P* < 0.0001 vs. the cholesterol crystal group.

Pyroptosis is a unique form of programmed cell death accompanied with inflammatory response, which is mediated by inflammasome and is dependent on the activation of caspase-1 [17]. The expression level of NLRP3 was detected with immunofluorescence (Figure 2(a)), and the inflammasome-associated protein levels were measured by Western blot after treatment with CC. As depicted in Figures 2(b) and 2(c), NLRP3 and ASC-1 were remarkably upregulated by CC, as well as the core component cleaved caspase-1. Conversely, colchicine treatment significantly blocked NLRP3 signal activity; the expression levels of NLRP3 and ASC-1 were nearly half compared with the CC group (Figures 2(b) and 2(c)). Moreover, colchicine did not significantly affect procaspase-1 protein expression but attenuated the expression of cleaved caspase-1 (p20 active forms) in response to CC, which was almost twice lower at the concentration of 10 nM (Figures 2(b) and 2(c)). It is well known that NLRP3 inflammasome is a macromolecule polyprotein complex that regulates the production of the IL-1 family [18]; activated caspase-1 subsequently cleaves IL-1*β* and IL-18 into their bioactive forms, as well as the effector of pyroptosis GSDMD [19]. We next determined the mRNA or protein expression levels of IL-1*β*, IL-18, and GSDMD in the endothelium; the results demonstrated that colchicine significantly inhibited CC-induced mRNA and protein expression of IL-18 and IL-1*β* (Figures 2(b), 2(c), 2(e), and 2(f)), particularly the mRNA level of GSDMD (Figure 2(d)). Besides, we detected the expression of several inflammatory factors using RT-PCR, and the mRNA levels of IL-6, IL-8, and MCP-1 decreased significantly in the presence of colchicine (Figures 2(g) and 2(i)).

### 3.3. Colchicine Plays an Antipyroptosis Role by Inhibiting Intracellular Oxidative Stress

**Figure 3:**
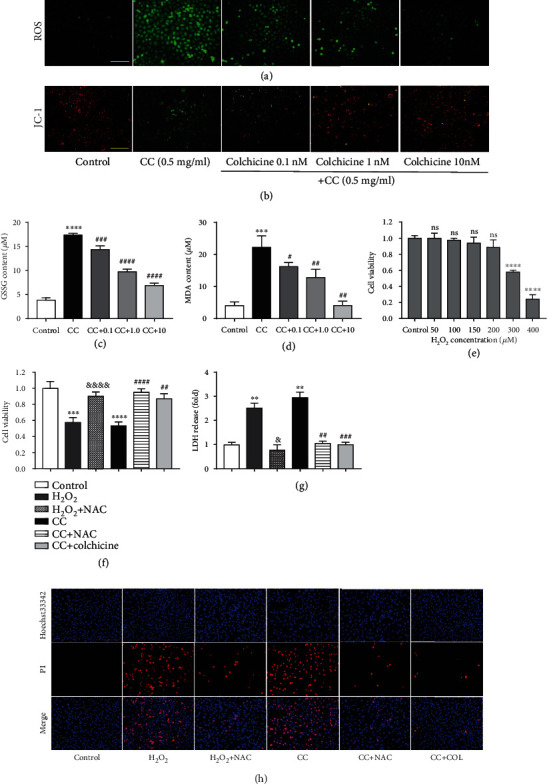
Colchicine plays an antipyroptosis role by inhibiting intracellular oxidative stress. HUVECs were treated for 10 or 24 h with different doses of colchicine (0–10 nM) in the presence of cholesterol crystal (0.5 mg/ml) or were left untreated (control). (a) Intracellular ROS level was detected using a DCFH-DA probe. (b) JC-1 Staining Kit was used for the detection of mitochondrial membrane potential. (c, d) The indicators of oxidative stress GSSG and MDA were tested by assay kits in endothelial cells. Endothelial cells treated with increasing doses of H_2_O_2_ (0-400 *μ*M); (e) the cell viability was detected using a CCK8 assay. HUVECs were subjected to H_2_O_2_ (300 *μ*M), cholesterol crystal (0.5 mg/ml) and colchicine (10 nM), or N-acetyl-cysteine (NAC, 5 mM) for 24 hrs. (f) The cell viability was detected using a CCK8 assay. Pyroptotic cell death was evaluated with (g) LDH release and (h) Hoechst 33342/PI staining. Scale bars = 100 *μ*m. Data was expressed as the mean ± SD of three separate experiments. ^∗∗^*P* < 0.01, ^∗∗∗^*P* < 0.001, and ^∗∗∗∗^*P* < 0.0001 vs. the control group. ^#^*P* < 0.05, ^##^*P* < 0.01, ^###^*P* < 0.001, and ^####^*P* < 0.0001 vs. the cholesterol crystal group. ^&^*P* < 0.05, ^&&&&^*P* < 0.0001 vs. the H_2_O_2_ group.

**Figure 4:**
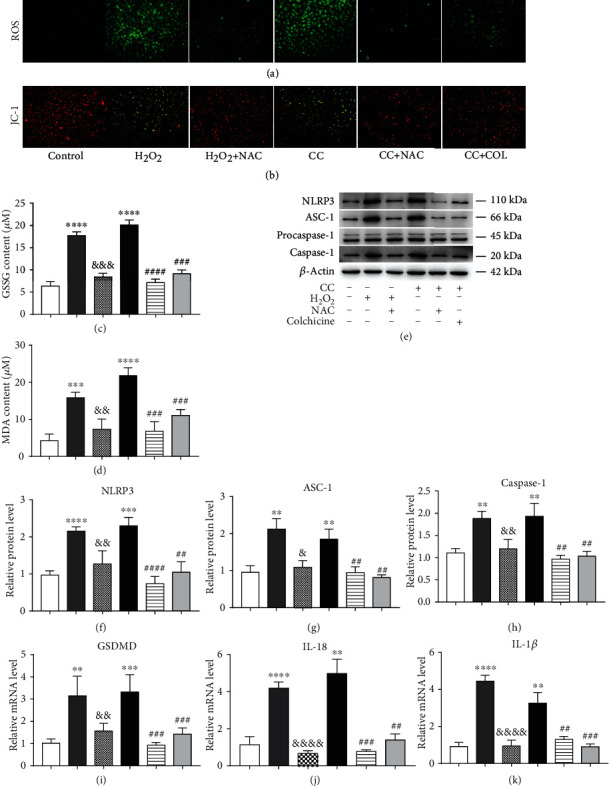
Colchicine plays an antipyroptosis role by inhibiting intracellular oxidative stress. HUVECs were subjected to H_2_O_2_ (300 *μ*M), cholesterol crystal (0.5 mg/ml) and colchicine (10 nM), or N-acetyl-cysteine (NAC, 5 mM) for 10 h or 24 h. (a) ROS level was detected using a DCFH-DA probe, and (b) mitochondrial membrane potential was tested by JC-1 (scale bars = 100 *μ*m). (c, d) The indicators of oxidative stress GSSG and MDA were tested by assay kits. (e) Western blotting was dedicated to examine the protein expression levels of NLRP3, ASC-1, procaspase-1, and caspase-1. (f–h) Quantitative analysis of pyroptosis-associated protein expression. (i, j) Real-time PCR analysis of the mRNA levels of GSDMD and proinflammatory cytokines (IL-18 and IL-1*β*). Data was expressed as the mean ± SD of three separate experiments. ^∗∗^*P* < 0.01, ^∗∗∗^*P* < 0.001, and ^∗∗∗∗^*P* < 0.0001 vs. the control group. ^##^*P* < 0.01, ^###^*P* < 0.001, and ^####^*P* < 0.0001 vs. the cholesterol crystal group. ^&^*P* < 0.05, ^&&^*P* < 0.01, ^&&&^*P* < 0.001, and ^&&&&^*P* < 0.0001 vs. the H_2_O_2_ group.

ROS generation is a well-validated mechanism for NLRP3 inflammasome activation [20]. Therefore, we first detected the changes of the ROS level in the endothelium treated with CC; our results demonstrated that CC treatment dramatically increased ROS levels, and this phenomenon was buffered by colchicine in a concentration-dependent manner (Figure 3(a)). Besides, we also detected a significant improvement in mitochondrial membrane potential, as well as a prominent reduction of the indicators of oxidative stress MDA and GSSG in the presence of colchicine (Figures 3(b)–3(d)). To further validate the mechanism underlying the suppression of pyroptosis by colchicine, we used a ROS scavenger N-acetyl-cysteine (NAC) and H_2_O_2_ as a positive control. Using CCK8, we found that the survival rate of endothelial cells decreased significantly when the H_2_O_2_ concentration exceeded 300 *μ*M (Figure 3(e)), and the cell viability was improved distinctly in the presence of NAC and colchicine (Figure 3(f)). The results in (Figures 3(g) and 3(h)) clearly indicated that NAC pretreatment could observably abrogate the release of LDH and the increase of PI-positive cells induced by CC and H_2_O_2_. Consistent results were also obtained by detecting the ROS level and the content of GSSG and MDA (Figures 4(a), 4(c), and 4(d)), while mitochondrial membrane potential improved significantly (Figure 4(b)). In addition, NAC also decreased CC and H_2_O_2_-induced protein expression levels of inflammasome components, including NLRP3, ASC, and cleaved caspase-1 (Figures 4(e)–4(h)); the gene expression of inflammatory cytokines, such as IL-18 and IL-1*β*, and the critical regulators GSDMD were all blocked by NAC, as well as colchicine at a concentration of 10 nM (Figures 4(i)–4(k)). These results demonstrated that colchicine attenuated CC-induced NLRP3 inflammasome activation and endothelial cell pyroptosis by reducing intracellular ROS overproduction and oxidative stress.

### 3.4. Colchicine Reverses the Downregulation of Various Antioxidant Enzymes and Promotes the Activation of AMPK-SIRT1 Signaling in Endothelial Cell

**Figure 5:**
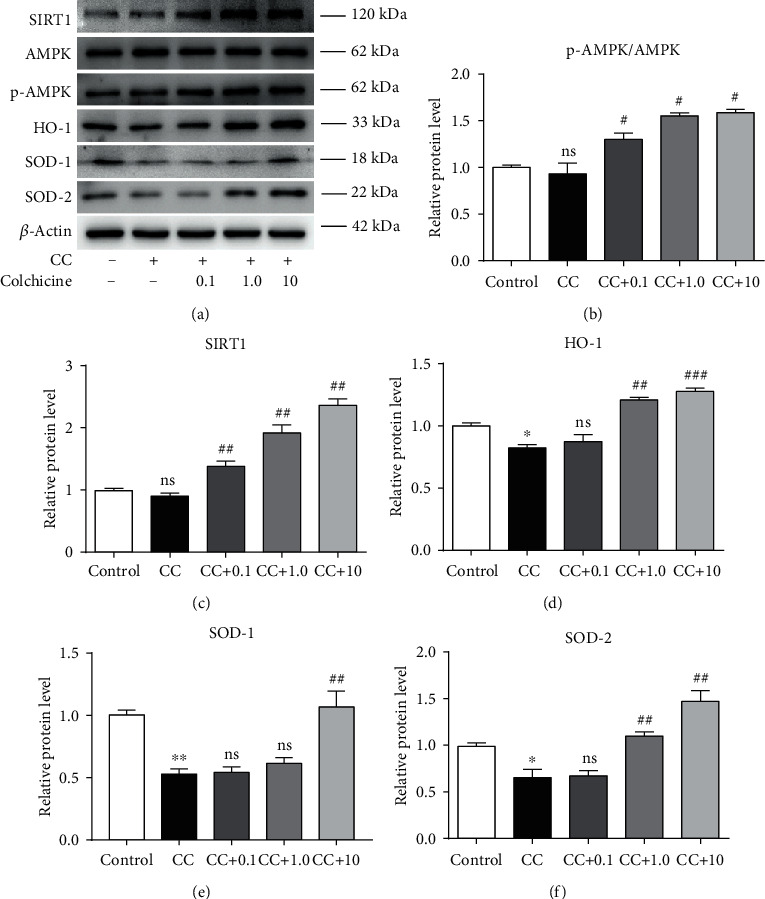
Colchicine reverses the downregulation of various antioxidant enzymes and promotes the activation of AMPK-SIRT1 signaling in endothelial cells. HUVECs were treated for 24 h with different doses of colchicine (0–10 nM) in the presence of cholesterol crystal (0.5 mg/ml) or were left untreated (control). (a) Western blotting was dedicated to examine the protein expression levels of SIRT1, p-AMPK*α*, AMPK*α*, HO-1, SOD-2, and SOD-1. (b–f) Quantitative analysis of AMPK/SIRT1 pathway-associated protein expression. Data was expressed as the mean ± SD of three separate experiments. ^∗^*P* < 0.05, ^∗∗^*P* < 0.01 vs. the control group. ^#^*P* < 0.05, ^##^*P* < 0.01, and ^###^*P* < 0.001 vs. the cholesterol crystal group.

The AMPK-SIRT1 pathway plays a crucial role in inhibiting oxidative stress via enhancing the expression of antioxidant enzymes during atherosclerosis [21–23]. Notably, decreased antioxidant enzyme levels were detected in the endothelial cell after CC exposure (Figures 5(a) and 5(d)–5(f)). To further understand the mechanism of the inhibitive role of colchicine in NLRP3 inflammasome activation and HUVEC pyroptosis, we measured the expression levels of AMPK-SIRT1 pathway-related proteins using Western blotting. Compared with HUVECs treated with CC alone, the protein levels of p-AMPK and SIRT1 were all activated by coincubation with 10 nM colchicine (Figures 5(a)–5(c)). Additionally, the presence of colchicine led to a much higher level of antioxidant enzyme expression, including HO-1, SOD-2, and SOD-1, and these effects were particularly obvious at the dose of 10 nM (Figures 5(a) and 5(d)–5(f)). Furthermore, previous experimental studies have proved that antioxidant enzymes can blunt ROS generation to a great extent and sequentially inhibit intracellular oxidation and endothelial cell dysfunction [24]. Therefore, it is plausible to suppose that colchicine may upregulate the expression and activity of various antioxidant enzymes and reduce the production of ROS via activating the AMPK-SIRT1 pathway, thus restraining the activation of NLRP3 inflammasome and endothelial cell pyroptosis induced by CC.

### 3.5. Silencing of AMPK-SIRT1 Signaling Eliminates the Protective Effect of Colchicine on Endothelial Cells

**Figure 6:**
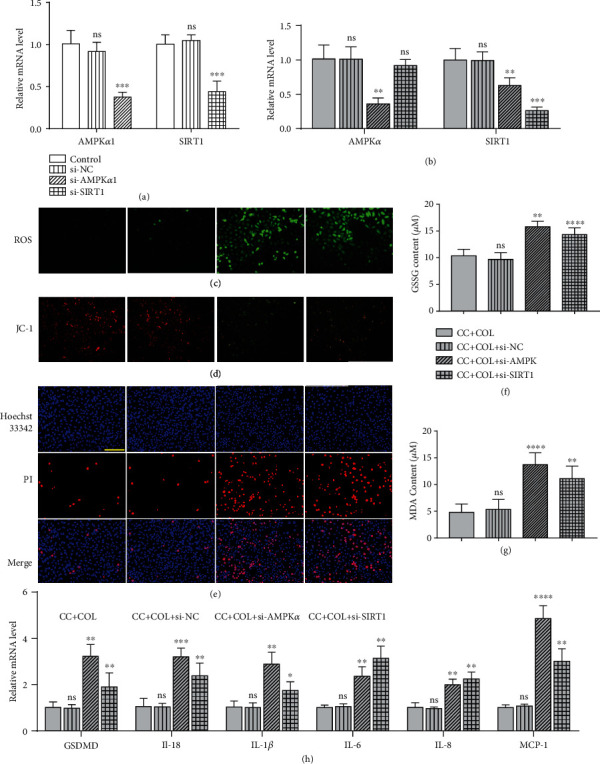
Silencing of AMPK-SIRT1 signaling eliminates the protective effect of colchicine on endothelial cells. HUVECs were transfected with siRNA targeting AMP*α*1 (si-AMPK*α*1) and SIRT1 (si-SIRT1) or a control siRNA (Si NC) or were not transfected (-). Cells were then added to cholesterol crystal (0.5 mg/ml) in the presence of colchicine (10 nM) for 10 or 24 hrs. Real-time PCR analysis of the mRNA expression of AMPK*α* and SIRT1 after transfection with si-AMPK*α*1, si-SIRT1, and si-NC for 24 hrs without (a) or with (b) the presence of cholesterol crystals and colchicine. (c) Intracellular ROS level was detected using a DCFH-DA probe, and (d) mitochondrial membrane potential was tested by JC-1. (e) Pyroptotic cell death was measured with Hoechst 33342 (blue)/PI (red) double-fluorescent staining. (f, g) The indicators of oxidative stress GSSG and MDA were tested by assay kits. (h) Real-time PCR analysis of the mRNA levels of GSDMD and proinflammatory cytokines (IL-18, IL-1*β*, IL-6, IL-8, and MCP-1). Scale bars = 100 *μ*m. Data was expressed as the mean ± SD of three independent experiments. ^∗^*P* < 0.05, ^∗∗^*P* < 0.01, ^∗∗∗^*P* < 0.001, and ^∗∗∗∗^*P* < 0.0001 vs. the si-NC group or CC+COL+si-NC group.

**Figure 7:**
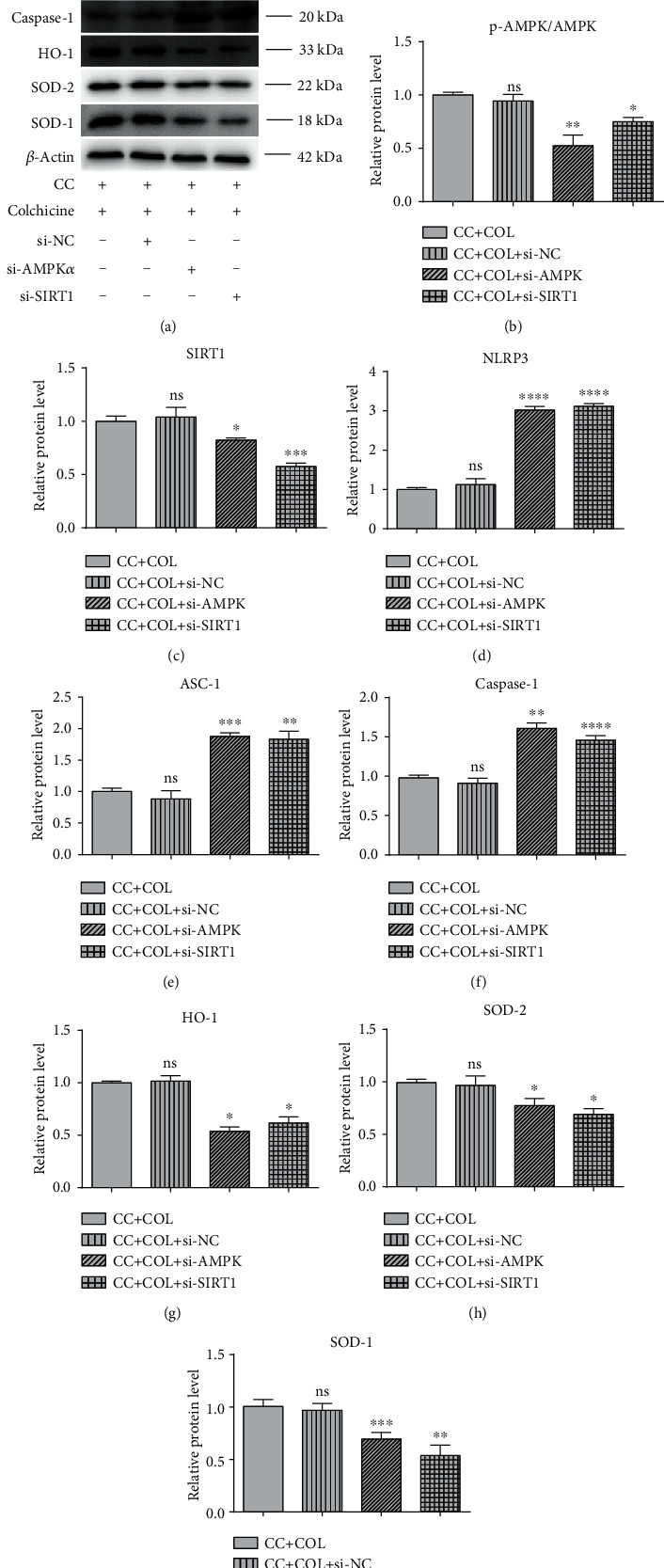
Silencing of AMPK-SIRT1 signaling eliminates the protective effect of colchicine on endothelial cells. HUVECs were transfected with siRNA targeting AMP*α*1 (si-AMPK*α*1) and SIRT1 (si-SIRT1) or a control siRNA (si-NC) or were not transfected (-). Cells were then added to cholesterol crystal (0.5 mg/ml) in the presence of colchicine (10 nM) for 24 hrs. (a) Western blotting was dedicated to examine the protein expression levels of SIRT1, p-AMPK*α*, AMPK*α*, HO-1, SOD-2, SOD-1, NLRP3, ASC-1, procaspase-1, and caspase-1 in endothelial cells. (b–i) Quantitative analysis of pyroptosis-associated protein and AMPK/SIRT1 pathway-associated protein expression. Values were expressed as the mean ± SD of three independent experiments. ^∗^*P* < 0.05, ^∗∗^*P* < 0.01, ^∗∗∗^*P* < 0.001, and ^∗∗∗∗^*P* < 0.0001 vs. the CC+COL+si-NC group.

**Figure 8:**
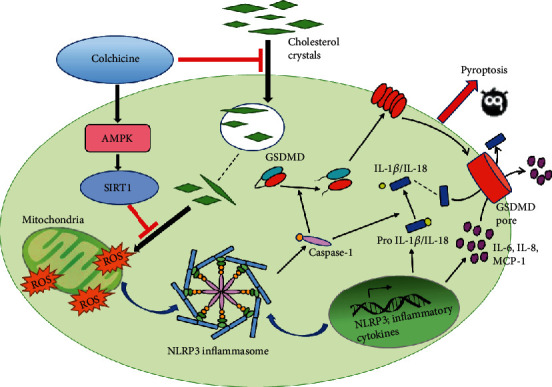
A model diagram showing the possible mechanism of colchicine alleviating cholesterol crystal-induced endothelial cell pyroptosis through activating the AMPK/SIRT1 pathway.

To further investigate whether colchicine's protective effect in HUVECs is AMPK-SIRT1 dependent, we carried out siRNA transfection experiment. The gene expression of AMPK*α*1 and SIRT1 was knocked down in endothelial cells using siRNA, respectively. To verify the silencing efficiency of siRNA, we analyzed the mRNA expression levels of AMPK*α*1 and SIRT1 in endothelial cells by RT-PCR. The results in Figures 6(a) and 6(b) demonstrated that transfection of si-AMPK*α*1 and si-SIRT1 significantly decreased the expression level of AMPK*α*1 and SIRT1 to more than 50% with or without the presence of cholesterol crystals and colchicine, whereas si-control (si-NC) had no obvious effect on HUVECs compared with the control group. Under the condition of CC and colchicine, AMPK*α*1 and SIRT1 knockdown induced a visible increase in the generation of ROS (Figure 6(c)) and the content of GSSG and MDA (Figures 6(f) and 6(g)), whereas there is an evident decrease of mitochondrial membrane potential (Figure 6(d)). Furthermore, compared with CC and colchicine stimulation, silencing of AMPK*α*1 and SIRT1 dampened the ability of colchicine to prevent HUVECs from CC-induced pyroptosis, as evidenced by the marked increase in the percentage of PI-positive cells (Figure 6(e)). Silencing of AMPK*α*1 and SIRT1 significantly suppressed the protein expression of NLRP3, ASC-1, and cleaved caspase-1 (Figures 7(a) and 7(d)–7(f)), along with the inhibition of the gene expression of proinflammatory cytokines (IL-1*β*, IL-18, IL-6, IL-8, and MCP-1) and GSDMD (Figure 6(h)). Consistent with previous studies [16], AMPK*α*1 silencing not only reduced the levels of AMPK*α*1 but also decreased that of SIRT1; transfection of si-SIRT1 only inhibited the expression of SIRT1 but did not significantly affect AMPK*α*1 protein expression, suggesting that AMPK may be a master upstream regulator of SIRT1 (Figures 6(b) and 7(a)–7(c)). Additionally, silencing of AMPK*α*1 and SIRT1 also remarkably abrogated the antioxidant enzyme expression even in the presence of colchicine, including HO-1, SOD-2, and SOD-1 (Figures 7(a) and 7(g)–7(i)). These findings confirmed that activating the AMPK-SIRT1 pathway and upregulating multiple antioxidant enzymes are at least partly relevant to the depression effect of colchicine on the inflammatory response and pyroptosis in HUVECs. A schematic summary of the proposed mechanism by which colchicine modulated the function of HUVECs is shown in Figure 8.

## 4. Discussion

The result presented in the current study demonstrated that colchicine plays a significant protective role in preventing the damage of endothelial cells induced by cholesterol crystals. Colchicine decreased cholesterol crystal-induced ROS generation in HUVECs and then inhibited NLRP3 inflammasome activation and inflammatory response, ultimately ameliorating the pyroptosis of endothelial cells. These effects suggest that colchicine has a promising role in the prevention and treatment of atherosclerosis.

A large number of autopsy evidences revealed that using optical coherence tomography (OCT), scanning electron micrographs, immunofluorescence, pathological staining, and other techniques could detect the dense concentration of cholesterol crystals perforating the fibrous cap and intima or depositing beneath the expansile fibrous cap in the coronary arteries of patients who died of acute coronary syndrome or acute myocardial infarction [2]. Therefore, as a crucial component of atherosclerotic plaque, cholesterol crystals play an important role in vulnerable plaque formation and subsequent cardiovascular adverse events [3]. Previous studies have shown that drugs such as ethanol and statins could dissolve preformed cholesterol crystals in atherosclerotic plaques [25], then provide plaque stabilization. Whereas our results unraveled that low doses of colchicine could inhibit the uptake of cholesterol crystals, while the concentration of colchicine was 0.1 nM, the content and volume of cholesterol crystals decreased significantly; also, the shape changed from sharp to blunt in endothelial cells. Moreover, after treatment with colchicine, endothelial cells containing cholesterol crystals can hardly be seen using TEM in all visual fields, especially at the concentration of 10 nM. This phenomenon indicates that colchicine could inhibit the phagocytosis of cholesterol crystals and even dissolve cholesterol crystals, which may contribute to the improvement of the stability of atherosclerotic plaques.

Duewell et al. provided the first evidence that in the early stage of atherosclerosis, small cholesterol crystals were abundant and simultaneously accompanied by the infiltration of inflammatory cells, which could activate NLRP3 inflammasome and promote the activation of caspase-1 and the release of mature IL-1*β* [26]. Subsequent studies have found that activation of inflammasome could not only induce secondary inflammatory responses but also result in pyroptotic cell death [11]. Pyroptosis is a recently discovered form of programmed cell death accompanied with inflammatory response, which has been implicated in the initiation and progression of atherosclerosis [27]. In agreement with the previous study, our experiments in human umbilical vein endothelial cells demonstrated that exogenous cholesterol crystal stimulation could lead to pyroptotic cell death. Using TEM, numerous pyroptotic cells were observed in all fields. These cells presented as condensed chromatin in the nucleus and cytoplasm swelling, lightening, or even rupturing. However, the protein holes formed on the surface of cell membranes were difficult to catch. In addition, the expression of pyroptosis-related proteins and the release of LDH were elevated significantly; on the contrary, the cell viability was obviously decreased. A study on gout shows that colchicine treatment significantly reduced MSU crystal-induced secretion of inflammatory cytokines (IL-1*β* and CXCL1) from macrophages and promoted macrophage differentiation into the anti-inflammatory M2 phenotype [15]. Since cholesterol crystals have the same physical and chemical properties with MSU crystals, both could activate NLRP3 inflammasome to abduct inflammatory response, which plays a causative role in inflammatory diseases. The previous study discovered that colchicine attenuated nonsteroidal anti-inflammatory drug-induced small intestinal injury by inhibiting the NLRP3 inflammasome activation [28]. Our in vitro results demonstrated that at the presence of colchicine, the pyroptotic cells were barely visible by TEM, along with decreased PI uptake, LDH release, and elevated cell viability. Meanwhile, the expression of NLRP3, ASC-1, and cleaved caspase-1 as well as the secretion of various inflammatory cytokines (IL-18, IL-1*β*, IL-6, IL-8, and MCP-1) was attenuated to varying degrees; the GSDMD N-terminal fragment was also reduced, especially at the concentration of 10 nM. These findings indicate that colchicine might be literally effective in suppressing the progress of atherosclerosis through inhibiting cholesterol crystal-induced endothelial cell pyroptosis.

Oxidative stress has direct damage to vascular parietal cells and is considered as a crucial driver in early atherogenesis [29]. The excessive accumulation of ROS, especially in mitochondria, plays an essential role in the occurrence of programmed cell death [30]. Previous studies highlighted that ROS is a vital upstream and positive regulator mediator in the activation of NLRP3 inflammasome [8, 31]. In our study, DCFH-DA probe detection shows that the stimulation of cholesterol crystals could induce ROS overproduction, along with the decrease of mitochondrial membrane potential, which was the same as H_2_O_2_. As major indicators of oxidative stress, GSSG and MDA levels were also significantly improved. Nonetheless, this consequence could be eliminated by NAC, a ROS scavenger, and colchicine had an equivalent effect. Moreover, NAC and colchicine pretreatment significantly reduced the elevated proportion of PI-positive cells and LDH release induced by cholesterol crystals; in addition, the expression of NLRP3-related proteins and inflammatory factors was also remarkably suppressed, which shows a powerful protective effect on endothelial cells. These results suggest that colchicine might repress pyroptotic cell death and NLRP3 inflammasome activation through inhibiting the generation of ROS induced by cholesterol crystals. This is also the first time that colchicine is confirmed to be effective in antioxidative stress and antipyroptosis beyond simple inflammation, indicating that colchicine has formidable potential in the cardioprotective role.

AMPK is a vital regulator of energy metabolism. In the prevention and treatment of AS, AMPK plays an important role in promoting cholesterol efflux, accelerating fatty acid oxidation and inhibiting inflammation [32–34]. Our results showed that colchicine treatment significantly increased the expression of phosphorylated AMPK and the downstream protein SIRT1. After silencing AMPK*α*, the expression levels of AMPK, p-AMPK, and SIRT1 were dramatically decreased. Whereas silencing of SIRT1 did not alter the expression of AMPK and p-AMPK, only the SIRT1 expression was downregulated significantly. These results confirmed that AMPK may be a pivotal upstream regulator of SIRT1. It has been shown that the activation of the AMPK/SIRT1 pathway could suppress the evolution of atherosclerosis by inhibiting endothelial cell oxidative stress and apoptosis, playing an effective and protective role in a variety of inflammatory-related disease [23, 35]. Our study also verified that colchicine could upregulate the expression of various antioxidant enzymes such as SOD-1, SOD-2, and HO-1 and reduce the production of ROS and the levels of GSSG and MDA, while mitochondrial membrane potential was improved significantly. Nevertheless, silencing of AMPK*α* and SIRT1 ameliorated colchicine-induced antioxidant enzyme upregulation, lower expression of ROS, downregulation of NLRP3 inflammasome-related proteins, and proinflammatory cytokines, suggesting that the specific mechanism of colchicine inhibiting endothelial cell pyroptosis might be tightly associated with the activation of the AMPK/SIRT1 pathway, which provides a strong theoretical basis for colchicine to become a promising therapeutic drug of atherosclerosis.

It is well known that the AMPK/SIRT1 pathway could modulate the function of vascular endothelial cells in diversiform ways, for instance, activating eNOS, PGC-1*α*, p53, Nrf2, and FoxO3 and inhibiting the activity of various inflammation-related proteins such as p38MAPK and NF-*κ*B pathway. Endothelial nitric oxide synthase- (eNOS-) derived nitric oxide (NO) has the properties of antihypertension, antithrombosis, antiatherosclerosis, and antiobesity [36]. Recent studies have shown that western diet-induced obesity could decrease the expression and activation of AMPK*α* and SIRT1, intensified aortic oxidative stress, increased related inflammatory responses, and diminished eNOS phosphorylation/activation and NO production, leading to endothelial stiffness, aortic fibrosis, and remodeling [37]. Metformin and resveratrol are both recognized AMPK/SIRT1 activators. It was found that long-term intake of fructose in rats could contribute to dysregulation of adipocytokine expression in perivascular adipose tissue (PVAT) and the loss of endothelium-dependent vasodilation, whereas AICA riboside (AICAR) stimulation could restrain gene expression of proinflammatory adipocytokines, facilitate eNOS phosphorylation in the aorta, and restore the loss of endothelium-dependent vasodilation via activating the AMPK/SIRT1 pathway. Meanwhile, oral administration of resveratrol and metformin could produce the same effect [38]. Our research also confirmed that the phosphorylation of eNOS was significantly influenced by the stimulation of cholesterol crystals in vitro, but pretreatment with colchicine could reverse this alteration and ameliorate endothelial dysfunction against inflammatory insult in an AMPK/SIRT1-interdependent manner (Figure S1 ). In addition, the AMPK/SIRT1 pathway can also exert anti-inflammatory, antioxidative stress, and antiapoptosis effects by upregulating the PGC-1*α* pathway. PGC-1*α* (peroxisome proliferator-activated receptor *γ* coactivator 1*α*) is a transcription coactivator of plentiful genes involved in energy management and mitochondrial biogenesis. In the rat model of myocardial ischemia/reperfusion injury, Tilianin could improve mitochondrial energy metabolism through AMPK/SIRT1/PGC-1*α* signaling, attenuate oxidative stress, significantly decrease the level of ROS and MDA, markedly alleviate myocardial infarction, evidently enhance myocardial pathological morphology, and reduce myocardial ischemia/reperfusion injury [39]. However, whether colchicine could also exert antioxidative stress via activating the PGC-1*α* pathway remains unknown; we need more experiments to prove it.

## 5. Conclusions

In conclusion, our present study provides the first evidence that colchicine could suppress the uptake of cholesterol crystals, promote the dissolution of cholesterol crystals in the endothelial cell, and inhibit endothelial cell inflammation and pyroptosis induced by cholesterol crystals. These effects may be achieved via activating the AMPK/SIRT1 pathway and inhibiting ROS production and oxidative stress. Therefore, colchicine is expected to become a potential drug for the treatment of atherosclerosis. However, further studies are required to validate whether colchicine could inhibit endothelial cell pyroptosis, thereby stabilizing atherosclerotic plaques and decreasing cardiovascular adverse events by activating the AMPK/SIRT1 pathway in vivo. Furthermore, the mechanism of colchicine inhibiting the uptake of cholesterol crystals still needs further experiments to explore.

## Data Availability

The data used to support the findings of this study are available from the corresponding author upon request.

## Supplementary Materials

Supplementary materials Figure S1: colchicine reverses the downregulation of eNOS phosphorylation by CC. HUVECs were treated for 24 h with different doses of colchicine (0–10 nM) in the presence of cholesterol crystal (0.5 mg/ml) or were left untreated (control). (a) Western blotting was dedicated to examine the protein expression levels of p-eNOS and eNOS. (b) Quantitative analysis of eNOS pathway-associated protein expression. HUVECs were transfected with siRNA targeting AMP*α*1 (si-AMPK*α*1) and SIRT1 (si-SIRT1) or a control siRNA (si-NC) or were not transfected (-). Cells were then added to cholesterol crystal (0.5 mg/ml) in the presence of colchicine (10 nM) for 24 hrs. (c) Western blotting was dedicated to examine the protein expression levels of p-eNOS and eNOS. (d) Quantitative analysis of eNOS pathway-associated protein expression. Data was expressed as the mean ± SD of three separate experiments. ^∗^*P* < 0.05, ^∗∗∗^*P* < 0.001 vs. the control group or CC+COL+si-NC group. ^###^*P* < 0.001 vs. the cholesterol crystal group
