# Supplementary material for: Colchicine Alleviates Cholesterol Crystal-Induced Endothelial Cell Pyroptosis through Activating AMPK/SIRT1 Pathway
Source: Oxid Med Cell Longev. 2020 Jul 15;2020:9173530. doi: 10.1155/2020/9173530 (PMC7378601; doi:10.1155/2020/9173530)
Supplement: Supplementary materials — Figure S1: colchicine reverses the downregulation of eNOS phosphorylation by CC. HUVECs were treated for 24 h with different doses of colchicine (0–10 nM) in the presence of cholesterol crystal (0.5 mg/ml) or were left untreated (control). (a) Western blotting was dedicated to examine the protein expression levels of p-eNOS and eNOS. (b) Quantitative analysis of eNOS pathway-associated protein expression. HUVECs were transfected with siRNA targeting AMPα1 (si-AMPKα1) and SIRT1 (si-SIRT1) or a control siRNA (si-NC) or were not transfected (-). Cells were then added to cholesterol crystal (0.5 mg/ml) in the presence of colchicine (10 nM) for 24 hrs. (c) Western blotting was dedicated to examine the protein expression levels of p-eNOS and eNOS. (d) Quantitative analysis of eNOS pathway-associated protein expression. Data was expressed as the mean ± SD of three separate experiments. ∗P < 0.05, ∗∗∗P < 0.001 vs. the control group or CC+COL+si-NC group. ###P < 0.001 vs. the cholesterol crystal group [file 9173530.f1.docx]

**Oxidative Medicine and Cellular Longevity**

**Colchicine alleviates cholesterol crystals induced endothelial cells pyroptosis through activating AMPK/SIRT1 pathway**

Mengyue Yang^1,2^, Qi Liu^1,2^, Lu Zhang^3^, Ruoxi Zhang^1,2^, Xingtao Huang^1,2^, Xuedong Wang^1,2^, Baihe Han^1,2^, Shenglong Hou^1,2^, Dandan Liu^1,2^, Gang Wang^1,2^, Jingbo Hou^1,2*^, Bo Yu^1,2^

^1^The Key Laboratory of Myocardial Ischemia Organization, Chinese Ministry of Education, Harbin, Heilongjiang, 150086, China;

^2^Department of Cardiology Organization, The Second Affiliated Hospital of Harbin Medical University, Harbin, Heilongjiang, 150086, China;

^3^Department of Cardiology, The Affiliated Cardiovascular Hospital of Xiamen University, Xiamen, Fujian, 316006, China

Correspondence should be addressed to Jingbo Hou; [Jingbohou@163.com](mailto:Jingbohou@163.com)

**Supplementary Figure1**


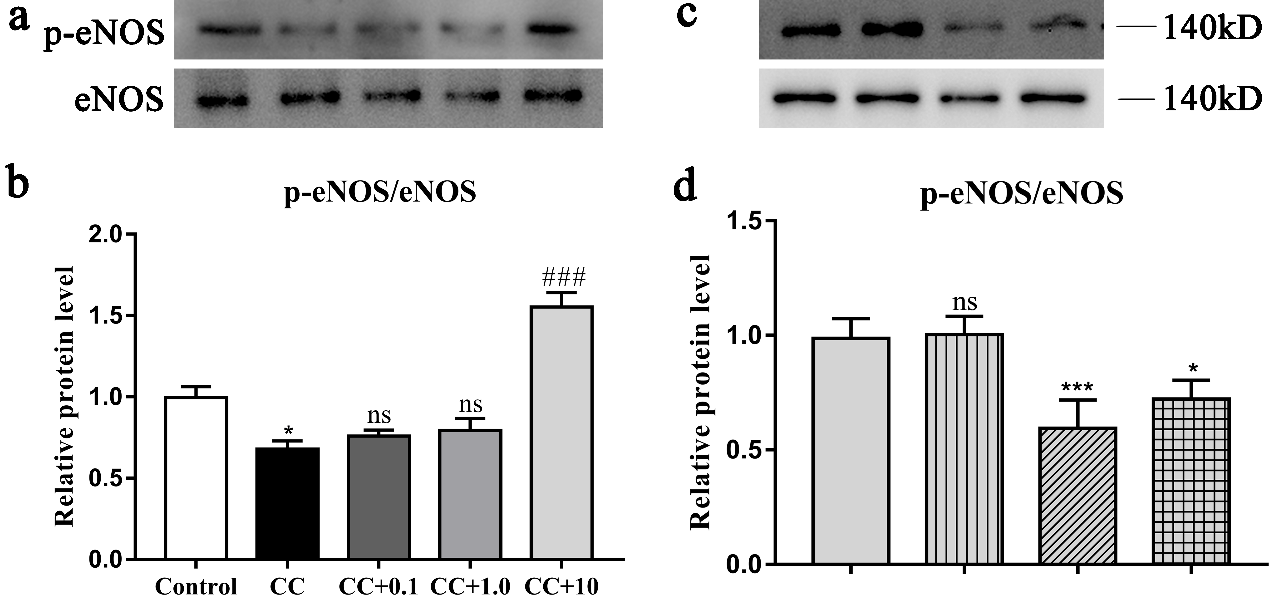


Figure S1. Colchicine reverses the downregulation of eNOS phosphorylation by CC. HUVECs were treated for 24h with different dose of colchicine (0–10nM) in the presence of cholesterol crystal (0.5mg/ml) or were left untreated (control). (a)Western blotting was dedicated to examine the protein expression levels of p-eNOS and eNOS. (b) Quantitative Analysis of eNOS pathway-associated protein expression. HUVECs were transfected with an siRNA targeting AMPα1 (Si AMPKα1) and SIRT1 (Si SIRT1), a control siRNA (Si NC), or were not transfected (-). Cells were then added to cholesterol crystal (0.5mg/ml) in the presence of colchicine (10nM) for 24 hrs. (c) Western blotting was dedicated to examine the protein expression levels of p-eNOS and eNOS. (d) Quantitative Analysis of eNOS pathway-associated protein expression. Data was expressed as mean ± SD of three separated experiments. *P < 0.05, ***P < 0.001, vs. the control group or CC + COL + Si NC group. ###P < 0.001 vs. the cholesterol crystal group.
